# Electrocardiographic features of the presence of occult myocardial disease in patients with VPD‐induced cardiomyopathy

**DOI:** 10.1002/joa3.12324

**Published:** 2020-03-05

**Authors:** Sung Il Im, Hye Bin Gwag, Youngjun Park, Seung‐Jung Park, June Soo Kim, Young Keun On, Kyoung‐Min Park

**Affiliations:** ^1^ Division of Cardiology Department of Internal Medicine Kosin University Gospel Hospital Kosin University College of Medicine Busan Republic of Korea; ^2^ Division of Cardiology Department of Medicine Samsung Medical Center Sungkyunkwan University School of Medicine Seoul Republic of Korea

**Keywords:** cardiomyopathy, electrocardiogram, ventricular premature depolarization

## Abstract

**Background:**

Frequent ventricular premature depolarizations (VPDs) can cause reversible cardiomyopathy (CMP). However, many patients maintain a normal left ventricular (LV) function with a high VPD burden. The electrocardiographic characteristics of VPD‐induced CMP have not been elucidated.

**Methods:**

One hundred and eighty (91 men, age; 51 ± 15 years) patients with frequent idiopathic VPDs (>10% VPDs/day or >10 000 VPDs/day) were studied. All patients underwent successful ablation and were then divided into two groups according to the echocardiographic findings before and after the ablation procedure.

**Results:**

Group A (n = 139) had a normal LV function with VPD frequencies, and Group B (n = 41) had reversible LV dysfunction after ablation. The VPD QRS duration (QRSd) was wider in patients with CMP (Group A vs Group B; 137.2 ± 12.0 milliseconds vs 159.7 ± 5.3 milliseconds, *P* < .001). VPDs with a terminal QRS delay marked by a notch followed by a discrete lower amplitude signal after the peak R wave in any precordial lead were identified. The incidence of terminal signals was higher in the CMP group (Group A vs Group B; 2.1% vs 53.6%, *P* < .001).

**Conclusions:**

The wider VPD QRSd and terminal QRS delay in patients with VPD‐induced CMP suggest subclinical cell‐to‐cell conduction abnormalities as a potential factor predisposing VPD‐induced CMP.

## Graphical abstract

Figure 1 Eletrocardiographic parameters. A, CI and post‐VPD CI; B, QR and RS interval and amplitude; C, r/R transition interval; D, Initial and terminal Angle of VPD. Abbreviations: CI, coupling interval; SCL, sinus cycle length; VPD, ventricular premature depolarization

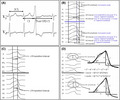

## 1 INTRODUCTION

Idiopathic ventricular premature depolarizations (VPDs) are usually considered a benign condition, even when the VPDs are frequent.^1^, ^2^ However, the VPD burden is one of the main causes leading to left ventricular (LV) dysfunction.^3^, ^4^ Previous several studies have described that a high burden of VPDs (>24%) is associated with LV cardiomyopathy (CMP) that resolves after successful VPD ablation.^4^, ^5^, ^6^, ^7^, ^8^ However, in 25%‐30% of patients the status of the LV systolic function cannot be explained only by the VPD burden.^9^ The mechanisms underlying the development of VPD‐induced CMP are not completely understood. In our experience, some VPD patients maintained normal ventricular function even with persistent ventricular bigeminy, while a significant number of patients had a depressed LV function, continuously, even with a lower VPD burden. Nishikawa et al reported that mild to severe interstitial fibrosis was observed by myocardial biopsy performed in patients with various arrhythmias.^10^ It could be that the extent of microscopic myocardial disease at baseline varies, which would not be detected by imaging studies in patients with idiopathic VPD‐induced CMP. In this study, we sought to find the useful electrocardiographic (ECG) characteristics of patients with VPD‐induced cardiomyopathy by analyzing and comparing the clinical and ECG parameters between patient groups with normal LV function and VPD‐induced CMP after undergoing successful radiofrequency catheter ablation (RFCA).

## 2 METHODS

### 2.1 Study population

A total of 282 patients underwent RFCA of VPDs at the Samsung Medical Center (SMC) from January 2008 to December 2016. All procedures were performed following the institutional guidelines of the University of Sungkyunkwan Health System and all patients provided written informed consent prior to participation as stipulated in the Declaration of Helsinki.

### 2.2 The inclusion criteria were as follows

All patients were enrolled based on the following criteria: (a) frequent VPDs (>10% or 10 000 VPDs/day); (b) no evidence of structural heart disease; (c) history of a successful VPD ablation (>80% suppression of the VPD burden); (d) baseline and follow‐up transthoracic echocardiography (TTE), 24 hours Holter monitoring data; (e) had no evidence of sustained ventricular tachycardia (VT); and (f) stopped all anti‐arrhythmic drug (AAD) use for at least five half‐lives.

And the patients with normal LV ejection fraction (EF) were defined as Group A. And those with depressed LV EF at baseline and with normalized LV EF after a successful VPD ablation were defined as Group B. Successful ablation was defined as having at least an 80% reduction in the 24‐hour burden of VPDs, based on our previously published experience.^11^ We did coronary angiogram or computed tomographic coronary angiogram for evaluation of coronary lesions in all patients.

Among 282 patients, 102 were excluded due to the following causes: episodes of sustained VT (n = 11); coronary artery disease (n = 10); LV non‐compaction (n = 1); sarcoidosis (n = 3); arrhythmogenic right ventricular cardiomyopathy/dysplasia (n = 2); myocarditis (n = 1); other magnetic resonance imaging (MRI) abnormalities (n = 3); procedure failure (n = 10); incomplete data (n = 42); and a depressed EF after a successful ablation (n = 19). One hundred and eighty patients (105 men, mean age; 51 ± 15 years) matched the inclusion criteria and were enrolled in the final study.

### 2.3 Assessment of the LV function

TTE was performed before the ablation procedure using the Simpson formula. For assessment of the LV EF, the second of three consecutive sinus beats was used to avoid any post‐extra‐systolic potentiation. An LV EF of <50% was considered abnormal. TTE with a quantitative assessment of the LV function was repeated 3‐6 months post‐ablation.

### 2.4 Follow‐up

Patients were seen in an outpatient clinic 3, 6, and 12‐48 months post‐ablation. 24 hours Holter monitoring was performed prior to the ablation procedure to measure the VPD burden (% and numbers/day). Follow‐up Holter monitoring was repeated 3‐6 months post‐ablation and again, later, if palpitations recurred. All anti‐arrhythmic drug therapy was discontinued if the ablation was effective. β‐Blockers(BB) and heart failure medications were continued initially and were discontinued if and when the LV function and dimensions normalized. No new medications were added after an effective ablation procedure.

### 2.5 ECG measurement

**FIGURE 1:**
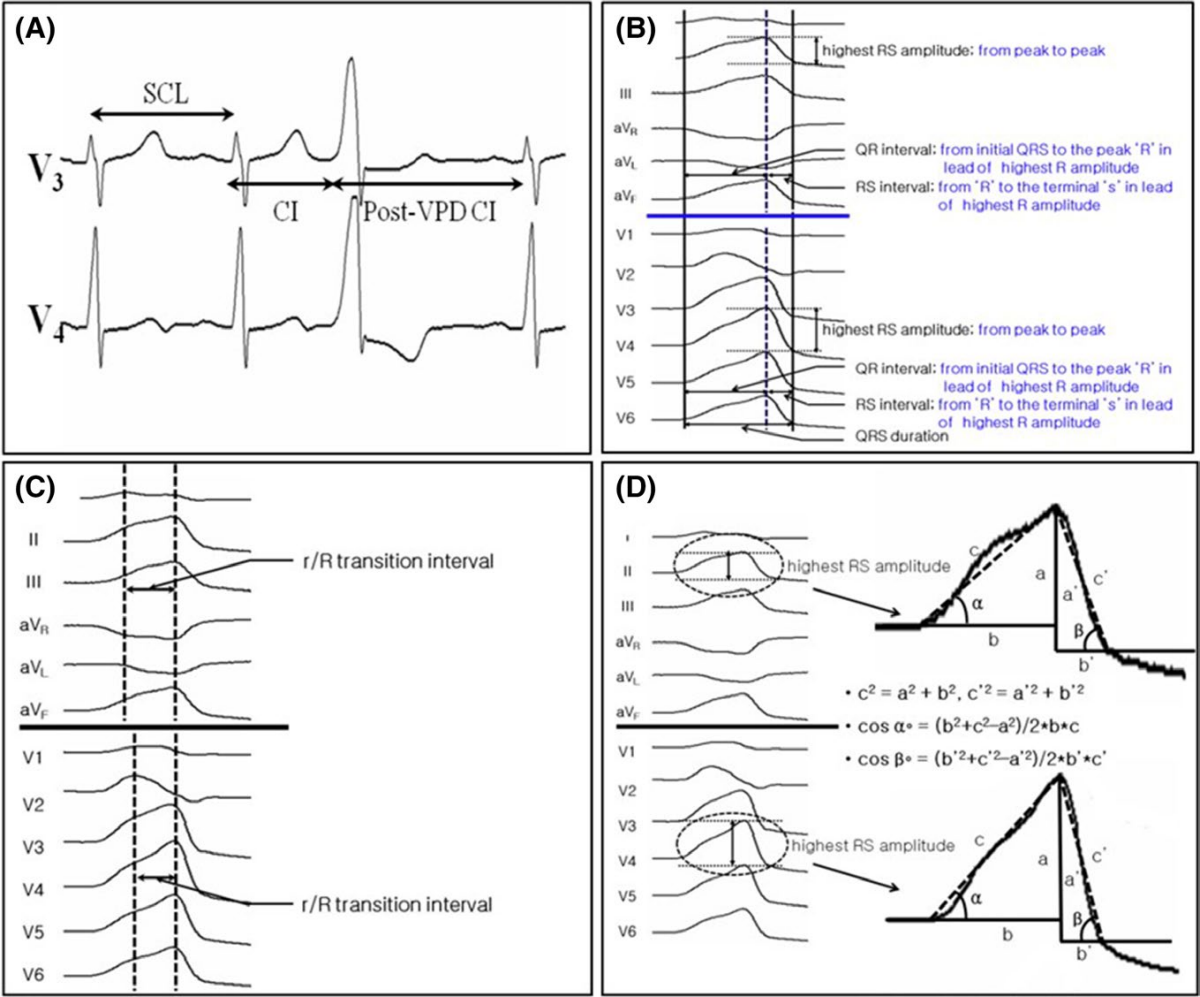
Eletrocardiographic (ECG) parameters. A, CI and post‐VPD CI; B, QR and RS interval and amplitude; C, r/R transition interval; D, initial and terminal angle of VPD. CI, coupling interval; SCL, sinus cycle length; VPD, ventricular premature depolarization

Sinus rhythm and the VPD ECG morphology were measured on the same 12‐lead ECG with electric calipers on the Prucka Cardiolab recording system (GE Healthcare; Figure 1). A standard 12‐lead ECG electrode placement was used. The lead gain was uniform with a paper speed of 100 mm/s. Additional clinical and electrophysiologic parameters were assessed by a detailed retrospective review. All electrocardiographic measurements were performed, blinded to the TTE outcomes, by one of the two authors (SII or KMP) using digital calipers at 100 mm/s, on the CardioLab® (version 6.5.4.1858; GE Medical Systems). To distinguish between the true J point and presence of retrograde p waves in the measurement of the VPD QRS duration (QRSd), we examined the intracardiac electrograms (after catheters were in place) to evaluate any retrograde conduction. Assessment of the interobserver variability was performed for the measurements of the VPD QRSd and VPD coupling interval (CI; Figure 1A‐C).

#### 2.5.1 Durations (width) and intervals

During the clinical VPD, the following measurements were obtained during both sinus rhythm (SR) and VPDs; (a) sinus cycle length (SCL); (b) sinus QRS width; (c) VPD QRSd; (d) CI between sinus rhythm and VPDs; (e) coupling interval ratio (CI/SCL); (f) post‐VPD CI between VPDs and sinus rhythm; (g) post‐VPD CI ratio (post‐VPD CI/ SCL); (h) qR width; (i) Rs width; (j) r/R wave transition interval; and (k) VPD notch duration.

The VPD QRSd was defined as the interval from the earliest ventricular activation to the offset of the QRS in any of the 12 leads. The CI was defined as the interval from the onset of the “q” or “R” wave of the previous sinus rhythm to the onset of the VPD QRS. The CI ratio was defined as the CI divided by the SCL. The post‐VPD CI was defined as the interval from the VPD onset to the onset of the “q” or “R” wave of the next sinus rhythm. The post‐VPD CI ratio was defined as the post‐VPD CI divided by the SCL. The “qR” width was defined as the width from the initial onset of the “q” wave of the VPD to the highest peak of the “R” wave in the precordial and limb leads. The “Rs” width was defined as the width from the peak “R” at the highest amplitude, in the same lead as the qR interval, to the terminal s wave in the precordial and limb leads. The “r/ R” transition interval was defined as the interval from the peak point of the earliest “r/ R” to the peak point of the latest “r/ R” in the precordial and limb leads. The definition of an “r” wave was one with an amplitude of over 0.1 mV. If there was notching in the summit of the VPD, the dominant peak was measured.

#### 2.5.2 Parameters

The following parameters were obtained during normal SR and VPDs: (a) highest “RS” amplitude on the precordial and limb leads; (b) maximal “R” and “S” amplitude on the precordial and limb leads; (c) initial and terminal angles measured using the Pythagorean theorem from the isoelectric line to the peak of the “R” or “S” wave in the lead with the highest amplitude in the precordial and limb leads (Figure 1D).

The highest RS amplitude was defined as the highest amplitude from the peak “r/ R” to the peak “s/ S” in the precordial and limb leads. The maximum “R” amplitude was defined as the amplitude from the maximum peak “R” point to the isoelectric line in the precordial and limb leads. The angles were measured using the Pythagorean theorem in the lead with the highest “R” amplitude. If there was no “R” wave in either the precordial or limb leads, then the “S” wave was used instead of the “R.” The initial angle was defined as the angle between the QRS onset to the peak “R” or “S” peak and the isoelectric line. The terminal angle was defined as the angle between the terminal point of the QRS to the peak “R” or “S” peak and the isoelectric line.

### 2.6 Terminal signals

**FIGURE 2:**
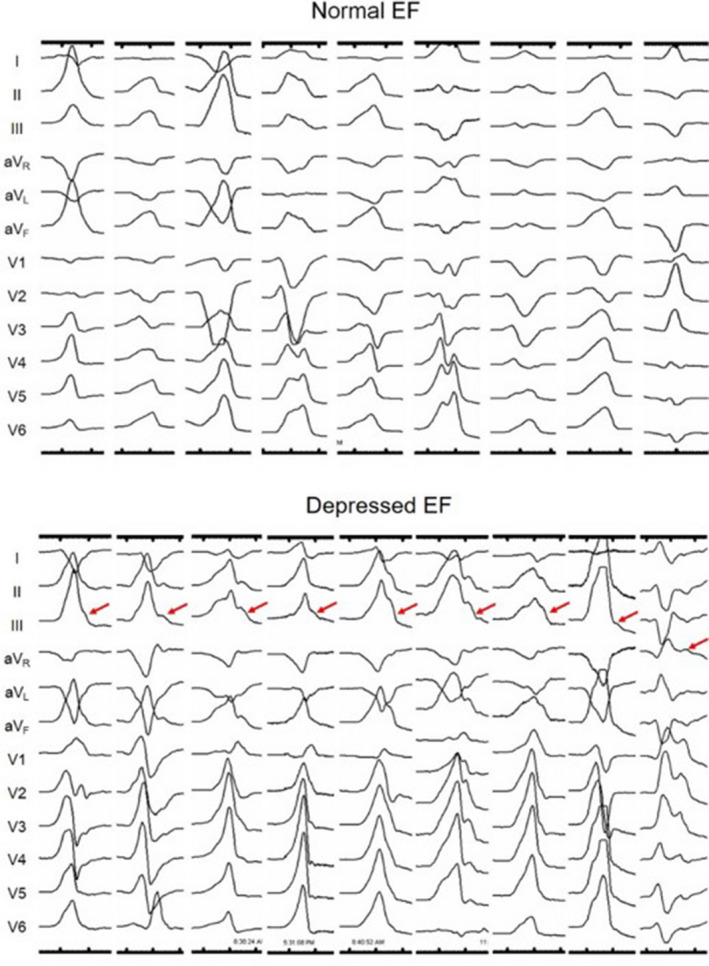
Twelve‐lead ECG showing the morphological features of the VPDs, suggesting a normal control (upper) and reversible CMP (lower). Terminal signal‐like morphologies are seen in patients with depressed EF (red arrow)

Terminal signals were defined as the terminal QRS delay marked by a notch followed by a discrete lower amplitude signal after the peak R wave in any precordial lead. If there were any potential‐like terminal signals in the precordial leads, we considered as those patients with terminal signals (Figure 2).

### 2.7 Statistical analysis

The Pearson's product moment correlation coefficient was calculated to quantify the inter‐variability in the measurement of both the VPD QRSd and coupling interval. Differences in the baseline characteristics across the groups of interest were carried out first in a univariate fashion using a Fisher's exact test for categorical variables and the Kruskal‐Wallis test for continuous variables. The percent of VPDs over 24 hours was a priori included in the preliminary main effects model given its clinically plausible influence on the outcomes following ablation. For all continuous variables, the linear assumption was tested by plotting the coefficients versus quartiles as well as performing a lowness smoothed plot of the continuous variables against the logit of the dependent variables. A goodness of fit was assessed by calculating the area under the curve of the receiver operating table and using the Hosmer‐Lemeshow test. All analyses were performed using SPSS Version 18.0 for Windows software (SPSS Inc). For all tests, a *P* < .05 was considered statistically significant.

## 3 RESULTS

Among a total of 180 patients with >10% VPDs/day (or >10 000 VPDs/day), 41 (23%) had an impaired LV function while the remaining 139 (77%) had a normal LV function and served as controls. Among the patients with an impaired LV function, 12 of 41 (29.2%) patients had failed prior attempts of ablation at referring institutions. An overall acute success rate, defined as no further appearance of clinical VPDs during a waiting period of at least 30 minutes after the ablation, was achieved in all cases (180; 100%). Among the patients with LV dysfunction, the rate for long‐term success for ablation, defined as a reduction of at least 80% in the VPD burden as determined by the post‐procedure Holter monitoring, was 82.9% (34/41), with second procedures (within our institution) required for success in seven (17%) of those patients. The median follow duration was 11.4 ± 7.5 months. Post‐procedure Holter monitoring was performed at a median of 1.6 months after ablation.

### 3.1 Baseline characteristics

There was no difference in both groups except age, gender, implanted cardiac defibrillator (ICD) implantation, use of BB, angiotensin‐converting enzyme inhibitor (ACEi), and angiotensin receptor blocker (ARB; Table 1). There are older patients in Group A (Group A; mean age; 49.9 ± 15.3 years vs Group B; 55.4 ± 14.5 years; *P* = .05). In Group B, male gender were enrolled more (A vs B; 48.2% vs 73.1%, *P* < .001). BB, ACEi, and ARBs were used more often by the patients in Group B than those in Group A (*P* < .001). The number of asymptomatic patients was significantly higher in Group B than in Group A (A vs B; 10% vs 41.4%, *P* < .001). In Group A, there was a higher incidence of palpitations associated with VPDs than in Group B (A vs B; 87% vs 39%, *P* < .001). The 24‐hour VPD burden (%) (A vs B; 21.6 ± 15.1% vs 29.0 ± 13.9%, *P* = .01) and absolute number of VPDs (A vs B; 23 754 ± 18 992 vs 34 979 ± 18 202, *P* = .005) were significantly higher in Group B. Among 180 patients, 28.9% underwent cardiac MRI and 13% underwent coronary angiography.

**TABLE 1:** Baseline epidemiologic and clinical characteristics in patients with ventricular premature depolarization (VPD) according to left ventricular dysfunction

	Group A N = 139	Group B N = 41	*P*‐value
Demographics			
Male sex, n (%)	67 (48.2%)	30 (73.1%)	<.001
Age, years	49.9 ± 15.3	55.4 ± 14.5	.05
BMI, kg/m^2^	28.9 ± 5.6	28.2 ± 6.1	.90
BSA, m^2^	2.01 ± 0.28	2.09 ± 0.31	.16
Medical history			
HTN, n (%)	38 (27.3%)	14 (34.1%)	.23
DM, n (%)	15 (10.8%)	5 (12.1%)	.77
ICD, n (%)	0 (0%)	5 (12.2%)	<.001
Atrial fibrillation, n (%)	12 (8.6%)	6 (14.6%)	.33
Medication history			
AAD^a^, (%)	24 (17.2%)	10 (24.3%)	.24
BB, n (%)	77 (54.6%)	33 (80.4%)	<.001
CCB, n (%)	23 (16.5%)	4 (9.7%)	.45
ACEI, n (%)	16 (11.5%)	12 (29.3%)	.005
ARB, n (%)	7 (5%)	8 (19.5%)	.004
Symptom history			
Asymptomatic	14 (10%)	17 (41.4%)	<.001
Symptom, n (%)	128 (92.1%)	21 (51.2%)	<.001
Palpitation, n (%)	121 (87%)	16 (39%)	<.001
SOB, n (%)	15 (10.8%)	1 (2%)	.20
Syncope, n (%)	19 (13.7%)	3 (7.3%)	.57
Dizziness, n (%)	22 (15.8%)	1 (2.4%)	.05
Fatigue, n (%)	9 (6.4%)	3 (7.3%)	.71
Symptom duration, m	64.2 ± 92.6	72.3 ± 95.9	.73
Holter monitoring			
VPD burden, %	21.6 ± 15.1	29.0 ± 13.9	.01
VPD burden, n	23 754 ± 18 992	34 979 ± 18 202	.005
NSVT, n (%)	53 (38.1%)	17 (41.4%)	.39
Multifocal VPDs, n (%)	12 (8%)	5 (12%)	.56
Echocardiography			
EF, %	59.3 ± 6.5	36.5 ± 7.6	<.001
LVEDD, mm	49 ± 6	54 ± 5	<.001
LVESD, mm	31 ± 5	42 ± 6	<.001
Cardiac MRI, n (%)			
Performed	39 (28%)	13 (31.7%)	0.67
Abnormal^b^	7 (5%)	6 (14.6%)	0.08

Abbreviations: AAD, anti‐arrhythmic drug; ACE, angiotensin‐converting enzyme; ARB, angiotensin receptor blocker; BB, β‐blocker; BMI, body mass index; BSA, body surface area; CCB, calcium channel blocker; DM, diabetes mellitus; HTN, hypertension; ICD, implanted cardiac defibrillator; LVEDD, left ventricular end‐diastolic dimension; LVESD, left ventricular end‐systolic dimension; LVEF, left ventricular ejection fraction; LV, left ventricle; MRI, magnetic resonance imaging; RV, right ventricle; m, months; NSVT, non‐sustained VT; SOB, shortness of breath; VPD, ventricular premature depolarization; Group A, normal EF; Group B, depressed EF.

^a^ Includes any class I or III anti‐arrhythmic drugs.

^b^ Defined as any area of delayed gadolinium enhancement or regional wall motion abnormality. The proportions presented are the number of abnormal exams over the number of subjects who underwent MRI.

### 3.2 ECG measurements

The sinus QRS width (A vs B; 87 ± 16 milliseconds vs 94 ± 18 milliseconds, *P* = .02) and VPD QRSd (A vs B; 137.2 ± 12.0 milliseconds vs 159.7 ± 5.3 milliseconds, *P* < .001) were significantly wider in Group B (Tables 2 and 3). The adjusted ratio between normal SR and the VPD width was also significantly higher in Group B (R vs N; 1.7 ± 0.2 vs 1.6 ± 0.2, *P* = .03). Within the VPD QRSd, the Rs intervals were prolonged significantly more in both the precordial (A vs B; 58.2 ± 14.7 milliseconds vs 78.0 ± 18.1 milliseconds, *P* < .001) and limb (A vs B; 57.5 ± 16.3 milliseconds vs 76.7 ± 15.8 milliseconds, *P* < .001) leads than the qR intervals in Group B as compared to Group A. Terminal peak angle was significantly higher in Group A than in Group B (precordial lead, *P* = .01; limb leads, *P* = .04), although there was no significant difference in the initial angle between Groups A and B.

**TABLE 2:** Comparison of the ECG parameters in patients with ventricular premature depolarization (VPD) according to left ventricular dysfunction

	Group A N = 139	Group B N = 41	*P*‐value
SCL, ms	814 ± 159	885 ± 176	.06
Sinus QRS width, ms	87 ± 16	94 ± 18	.02
VPD QRS width, ms	137.2 ± 12.0	159.7 ± 5.3	<.001
VPD/sinus QRS ratio	1.6 ± 0.2	1.7 ± 0.2	.02
CI, ms	503.3 ± 72.5	519.0 ± 75.5	.39
CI ratio	59 ± 11	61 ± 15	.31
VPD CI, ms	1085 ± 253	1141 ± 257	.53
VPD CI ratio	134 ± 24	130 ± 20	.72
Highest R amp (pre), mV	2.5 ± 0.7**^†^**	2.5 ± 1.4	.58
Highest R amp (limb), mV	1.7 ± 0.6	1.9 ± 0.7	.06
VPD notch duration, ms	29.0 ± 10.4	37.6 ± 26.6	.45
Terminal potential, n (%)	3 (2.1%)	22 (53.6%)	<.001
Sites of origin (RV), n (%)			
RVOT/RCC/PA	60 (43.1%)	9 (21.9%)	.09
Other RV	5 (3.6%)	1 (2.4%)	.87
LCC/AMC/RLJ/AIV	51 (36.7%)	16 (39%)	.16
Other LV	26 (18.7%)	12 (29.3%)^†^	.04
Multifocal VPDs, n (%)	12 (8.6%)	5 (12.2%)	.56
VPD site (1)^a^			
Septal	100 (71.9%)	20 (48.8%)	.14
Non‐septal	35 (25.2%)	14 (34.1%)	
VPD site (2)^a^			
Outflow tract	97 (69.7%)	19 (46.3%)	.08
Non‐outflow tract	38 (27.3%)	15 (36.6%)	

Abbreviations: CI, coupling interval; EF, ejection fraction; ms, msec; pre, precordial; SCL, sinus cycle length; VPD, ventricular premature depolarization; LV, left ventricle; ms, millisecond; RVOT, right ventricular outflow tract; RCC, right coronary cusp; PA, pulmonary artery; RV, right ventricle; LCC, left coronary cusp; AMC, aortomitral continuity; CC, coronary cusp; AIV, anterior interventricular vein; Group A, normal EF; Group B, depressed EF.

^a^ Subjects with multiple PVC morphologies were excluded.

**TABLE 3:** Comparison of ECG parameters in patients with ventricular premature depolarization (VPD) according to left ventricular dysfunction

	Group A N = 139	Group B N = 41	*P*‐value
qR width (pre), ms	79.3 ± 16.3	84.0 ± 21.4	.06
qR width (limb), ms	79.5 ± 16.2	85.4 ± 16.7	.07
Rs width (pre), ms	58.2 ± 14.7	78.0 ± 18.1	<.001
Rs width (limb), ms	57.5 ± 16.3	76.7 ± 15.8	<.001
r/R TI (pre‐NSR), ms	19.6 ± 11.7	22.9 ± 11.6	.18
r/R TI (limb‐NSR), ms	20.6 ± 11.3	20.2 ± 22.2	.71
r/R TI (pre‐VPD), ms	44.8 ± 19.4	45.6 ± 22.9	.63
r/R TI (limb‐VPD), ms	30.6 ± 13.8	30.7 ± 26.4	.82
Transition ratio (pre lead)	1.9 ± 1.3	2.8 ± 2.6	.08
Transition ratio (limb)	2.2 ± 1.8	2.2 ± 2.1	.51
Initial peak angle (pre), °	47.0^°^	50.0^°^	.16
Initial peak angle (limb), °	41.0^°^	41.0^°^	.87
Terminal peak angle (pre), °	51.5^°^	46.0^°^	.01
Terminal peak angle (limb), °	56.0^°^	51.5^°^	.04

Abbreviations: EF, ejection fraction; ms, msec; NSR, normal sinus rhythm; pre, precordial; VPD, ventricular premature depolarization; TI, transition interval; Group A, normal EF; Group B, depressed EF.

*P*‐value between irreversible and reversible CMP.

#### 3.2.1 Interobserver reliability of the ECG measurements

The correlation coefficient for the measurements of the VPD QRSd was 0.871, and that for the VPD coupling interval was 0.936.

### 3.3 Terminal signals

Potential‐like signals were found at the terminal portion of the clinical VPDs in three patients in Group A (2.1%) and 22 in Group B (53.6%; *P* < .001). Among the 22 patients in Group B, three had a past history of reversible CMP. Among the three patients in Group A, two had a borderline (EF = 50%) LV function (Figure 2).

## 4 DISCUSSION

### 4.1 Main findings of the study

It is increasingly recognized that idiopathic VPDs may cause LV dysfunction that is reversible after a successful ablation.^5^, ^9^ Recent studies suggest that a VPD frequency of more than 24% during 24‐hour Holter monitoring is a risk factor for VPD‐induced CMP.^3^, ^6^, ^7^, ^12^ However, 20%‐25% of the VPD patients did not meet this cutoff value in those studies. In fact, many patients maintained a normal LV function even with a high VPD burden. Inversely, some patients have reduced ventricular function with a lower VPD burden. One study to date has examined the longitudinal impact of VPD burden on the LV function, and a subclinical deterioration in the LV function was found with a high burden of VPDs over 5 years.^13^ Finally, the paradigms of VPD‐induced CMP could not be explained with the VPD burden alone. Recently, some authors have questioned the pre‐existence of occult structural heart disease as one of the mechanisms of VPD‐induced CMP.^13^, ^14^

Interestingly, in this study, the number of asymptomatic patients was significantly higher in Group B than in Group A (A vs B; 10% vs 41.4%, *P* < .001). This finding is consistent with previous study that a lack of symptoms could be associated with a greater risk of VPC‐induced cardiomyopathy.^15^, ^16^

In this study, we examined the clinical and electrocardiographic features in patients with VPD‐induced CMP compared with normal control patients. The VPD QRS duration of the CMP patients was significantly more prolonged than that of the normal patients, even after adjusting for the sites of origin of the VPDs, LV dimension, and body surface area. Among the ECG parameters, Rs width was significantly wider in Group B. Furthermore, only terminal peak angle was significantly lower in Group B. We also found abnormal distorted potential‐like signals within the Rs segment of the VPD, and were found predominantly more often in patients with LV dysfunction than in the normal control patients. From these results, we could infer carefully that Rs segment of VPD is more important to identify the presence of occult myocardial disease in the patients with VPD‐induced CMP than qR segment.

Several investigators have found potential contributing factors to explain the ventricular arrhythmias from endomyocardial biopsies or autopsies.^17^, ^18^, ^19^ Lemery et al^20^ described the clinical, laboratory, and electrophysiological features in idiopathic VT patients who had no clinical evidence of heart disease. This group reported that minor structural cardiac abnormalities were found in more than 30% of these patients. Nishikawa et al also mentioned that the advanced histopathologic findings, including myocyte hypertrophy, degeneration, interstitial fibrosis, and disarrangement of muscle bundles were observed in patients with idiopathic VT.^10^ Most imaging studies have not found any significant abnormalities in this patient population to corroborate the evidence of occult structural heart disease. However, current imaging technology might not be sensitive enough to identify all occult structural abnormalities that may predispose a patient to increased manifest CMP when the system is stressed. Some papers described that the VPD QRSd was significantly wider in patients with VPD‐induced CMP than in normal controls.^15^, ^21^ The wider VPD QRSd means a prolongation of the myocardial cell‐to‐cell conduction time and this finding may be indirect evidence of abnormal myocardial cells, such as those that are hypertrophied or fibrotic.

We also found nonspecific distorted abnormal potential‐like signals at later portions of the VPDs, predominantly in patients with CMP. Several previous reports have described the epsilon potential that occurs frequently in lead V_1‐2_ during sinus rhythm. This potential has been viewed as indirect evidence of RV myocardial dysplasia^22^, ^23^, ^24^, ^25^, ^26^, ^27^ and Brugada syndrome.^28^ And Moulton et al found a useful ECG marker for the left ventricular structure and function.^29^ They explained that a broadly notched VPD with a long duration was a useful marker for a dilated globally hypokinetic left ventricle. They described the mechanisms of this distorted VPD as dilatation of the T‐tubule system due to altered microanatomy and as an abnormality in the cell‐to‐cell communication by desmosomes. Even though 61% of the patients had coronary artery disease, it was a meaningful result in that the prolonged VPD QRS duration and notch on the ECG were important as indirect evidence of the myocardial status. Aizawa Y et al also reported that tachycardia‐dependent augmentation of "notched J waves" in a general patient population without ventricular fibrillation or cardiac arrest was augmented at shorter RR intervals, but not at prolonged RR intervals. Mechanistically, conduction delay is most likely responsible for this change.^30^ However, we do not know the meaning of these signals in our study and could not perform myocardial biopsy to confirm the results in the tissue. However, the signals occurred more frequently in CMP patients than in normal patients. These findings may suggest some occult microscopic myocardial disease in the myocardium. If the occult microscopic myocardial disease was distributed diffusely, a myocardial conduction delay would occur globally and the VPD QRSd would be significantly wider than normal. Whatever the mechanisms of these signals, a delayed slow potential or dyssynchrony, these results suggest a new direction for the electrophysiological and pathophysiological mechanisms that lead to VPD‐induced CMP.

### 4.2 Study limitations

There were some limitations in this study, First, this study was a single‐center, retrospective study derived from a real‐world practice with inherent limitations and we could not assess quantitative analysis of dyssynchrony by echocardiography. Hence the results of our study should be considered as hypothesis generating, and future prospective studies are warranted to confirm our results. Second, in our study, among all enrolled patients, 36% of the patients underwent cardiac MRI while 13% underwent CAG to rule out any structural heart disease. Therefore, we could not rule out with certainty the existence of minimal structural heart disease that could be detected by using TTE. Third, the accurate measurement of the LV dysfunction may have been compromised by frequent VPDs, particularly in patients with incessant ventricular bigeminy, who never have two simultaneous sinus beats.

## 5 CONCLUSION

In patients with idiopathic VPDs, the presence of wider VPD QRS duration and potential‐like signals at the terminal portion of VPD may be indirect evidence of the pre‐existence of occult microscopic myocardial disease with reversible CMP.

## CONFLICT OF INTERESTS

The authors declare no conflict of interests for this article.
